# Supplementary material for: Effect of Permissive Underfeeding with Intensive Insulin Therapy on MCP-1, sICAM-1, and TF in Critically Ill Patients
Source: Nutrients. 2019 Apr 30;11(5):987. doi: 10.3390/nu11050987 (PMC6566807; doi:10.3390/nu11050987)

Table S1: Baseline characteristic of all patients and with stratification by permissive underfeeding and target feeding groups and intensive insulin therapy and the conventional insulin therapy groups

Variable	Standard feeding		Permissive underfeeding		
	IIT + Standard feeding n = 24	CIT + Standard feeding n = 21	IIT + Permissive underfeeding n = 24	CIT + Permissive underfeeding n = 22	P-value
**Age (yrs),** mean ±SD	53±21	51±22	50±19	51±23	0.98
**Height (cm),** mean ±SD	164±12	161±12	164±12	167±8	0.37
**Weight (kg),** mean ±SD	73±20	77±21	80±19	79±17	0.54
**Body mass index,** mean ±SD	26.9±7.2	30.2±9.6	30.0±8.9	28.3±6.8	0.52
**Inclusion blood sugar at baseline (mmol/L),** mean ±SD	11.9±4.6	11.3±4.1	11.5±4.3	13.2±5.2	0.60
**APACHE II,** mean ±SD	27±7	25±9	25±8	25±7	0.57
**SOFA-Day1,** mean ±SD	11±3	10±3	10±3	10±3	0.65
**Creatinine (μmol/L),** mean ±SD	202±231	177±178	130±80	122±74	0.75
**Platelets x 10^9/L,** mean ±SD	236±158	217±136	180±103	25±186	0.49
**INR,** mean ±SD	1.3±0.4	1.3±0.3	1.6±1.0	1.3±0.3	0.56
**PaO_2:FIO_2,** mean ±SD	216±111	191±90	213±121	180±120	0.52
**GCS,** mean ±SD	7±3	9±4	7±2	8±4	0.47
**Gender (Female),** no(%)	9 (37.5)	8 (38.1)	9 (37.5)	4 (18.2)	0.41
**Diabetes,** no (%)	8 (33.3)	8 (38.1)	11 (45.8)	9 (40.9)	0.85
**Vasopressor,** no (%)	16 (66.7)	11 (52.4)	13 (54.2)	17 (77.3)	0.28
**Sepsis,** no (%)	8 (33.3)	4 (19)	5 (20.8)	7 (31.8)	0.59
**Admission category,** no (%)					

Medical	17 (70.8)	14 (66.7)	13 (54.2)	12 (54.5)	
Non-operative trauma	3 (12.5)	4 (19)	6 (25)	5 (22.7)	0.88
Post-operative	4 (16.7)	3 (14.3)	5 (20.8)	5 (22.7)	

P-value calculated using ANOVA, Kruskal Wallis or Chi-square as appropriate. BMI, body mass index; APACHE II, Acute Physiology and Chronic Health Evaluation II; SOFA, Sequential Organ Failure Assessment; INR, International Normalized Ratio; PaO_2:FIO_2 ratio, the ratio of partial pressure of oxygen to the fraction of inspired oxygen; GCS, Glasgow Coma Scale; ICU, intensive care unit; SD, Standard Deviation. To convert to conventional units in mg/dL, divide by 0.0555 for glucose, 88.4 for creatinine and 17.1 for bilirubin.

Table S1: Caloric intake, protein intake, insulin, and glucose and outcome data in the permissive underfeeding and target feeding groups and in the intensive insulin therapy and the conventional insulin therapy groups.

Variable	Standard feeding		Permissive underfeeding		
	**IIT + Standard feeding n = 24**	**CIT + Standard feeding n = 21**	**IIT + Permissive underfeeding n = 24**	**CIT + Permissive underfeeding n = 22**	**P-value[1]**
Calculated caloric requirement (kcal/day), mean ±SD	1692±257	1748±308	1802±300	1881±350	0.24
Study caloric target (kcal/day), mean ±SD	1675±240	1650±438	1272±213	1376±235	< 0.01
Average daily caloric intake (kcal/day), mean ±SD	1348.9±408.3	1405.0±416.2	1065.6±243.9	1154.3±293.0	< 0.01
Percent caloric intake/requirement (%), mean ±SD	79.71±21.9	80.3±17.6	59.56±11.1	62.8±15.9	< 0.01
Calculated protein requirement (gm/day), mean ±SD	70.3±12.4	71.8±14.3	75.0±21.6	81.8±12.3	0.08
Average daily protein intake (gm/day), mean ±SD	46.5±18.8	50.6±16.9	46.8±20.3	55.9±15.5	0.26
Average enteral calories, mean ±SD	1156.4±461.6	1245.8±391.1	904.6±333.3	1011.1±256.4	0.01
Average daily propofol calories (kcal)	50.5±104.0	84.8±161.0	46.6±84.2	59.0±94.5	0.98
Average daily dextrose calories (kcal), mean ±SD	141.9±165.1	74.4±96.4	115.0±111.0	84.1±99.2	0.13
Average daily insulin dose (Units), mean ±SD	66.6±39.8	24.7±41.4	69.1±53.1	33.6±47.4	< 0.01
Average glucose levels (mmol/L), mean ±SD	6.4±1.1	8.9±1.7	6.3±0.8	8.9±2.0	< 0.01
28-day mortality, no (%)	5 (20.8)	4 (19.0)	1 (4.2)	8 (36.4)	0.06
180-day mortality, no (%)	8 (33.3)	6 (28.6)	6 (25.0)	10 (47.6)	0.41
ICU mortality, no (%)	4 (16.7)	5 (23.8)	3 (12.5)	6 (27.3)	0.59
Hospital mortality, no (%)	9 (37.5)	6 (28.6)	6 (25.0)	10 (45.5)	0.47
Renal replacement therapy, no (%)	3 (12.5)	2 (9.5)	5 (20.8)	2 (9.1)	0.61
Hospital LOS (day), mean ±SD	90±105	93±80	97±108	74±95	0.45
ICU LOS (day), mean ±SD	16.3±11.2	17.1±13.9	13.7±8.5	12.9±6	0.81
Mechanical ventilation duration (day), mean ±SD	14±10.0	16±14.0	12±8.0	12±6	0.72

P-value calculated using ANOVA or Kruskal Wallis as appropriate. IIT: Intensive insulin therapy, CIT: Conventional insulin therapy, LOS: length of stay; SD: Standard Deviation.

Table S3: Comparison of inflammatory mediators/biomarkers by randomization at each point of time

Markers	Time	Standard feeding		Permissive underfeeding		P value
		IIT + Standard feeding	CIT + Standard feeding	IIT + Permissive underfeeding	CIT + Permissive underfeeding	
MCP-1 (pg/mL) Median (Q1,Q3)	Baseline	194 (130, 401)	225 (129,346)	237 (154,366)	382 (184,758)	0.18
	Day 3	218 (118,375)	154 (105,228)	184 (110,267)	218 (115,478)	0.44
	Day 5	153 (101,212)	141 (99,303)	128 (86,244)	137 (70,348)	0.96
	Day 7	113 (74, 246)	126 (78,247)	195 (92,265)	204 (118,322)	0.47
sICAM-1 (ng/mL) Median (Q1,Q3)	Baseline	279 (196,359)	228 (168,415)	234 (156,342)	239 (183,379)	0.9
	Day 3	299 (201,365)	245 (163,360)	248 (159,330)	237 (198,350)	0.72
	Day 5	311 (252,365)	245 (176,302)	220 (169,320)	214 (173,389)	0.21
	Day 7	300 (243,385)	280 (179,349)	246 (221,268)	261 (192,336)	0.47
TF (pg/mL) Median (Q1,Q3)	Baseline	73 (32,135)	60 (12,103)	33 (24,139)	63 (29,264)	0.43
	Day 3	58 (22, 132)	72 (14,107)	49 (23,99)	51 (31,250)	0.9
	Day 5	89 (48,131)	83 (24,115)	49 (31,120)	51 (23,267)	0.96
	Day 7	71 (23,123)	64 (20,83)	58 (34,121)	64 (31,274)	0.71

P-value calculated using ANOVA and Kruskal Wallis as appropriate. MCP-1: monocyte chemoattractant protein-1, sICAM-1: soluble intercellular adhesion molecule-1, TF: tissue factor

Table S4: Test variance-covariance matric structure.

Outcome	-2 Log Likelihood			P-value
	Diagonal	Unstructured	Difference	
MCP-1 (pg/mL)	3857.2 (df =11)	3720.2 (df =17)	137.0 (df =6)	< 0.01
sICAM-1 (ng/mL)	3346.5 (df =13)	3173.0 (df =19)	173.5 (df =6)	< 0.01
TF (pg/ml)	3867.9 (df =12)	3096.3 (df =18)	771.6 (df =6)	< 0.01

df :degree of freedom

Figure S1: Caloric intake, protein intake, insulin, and glucose and outcome data in the permissive underfeeding and target feeding groups and in the intensive insulin therapy and the conventional insulin therapy groups.

**Intensive insulin therapy (IIT) vs. conventional insulin therapy (CIT)**	**Permissive underfeeding vs. target feeding**
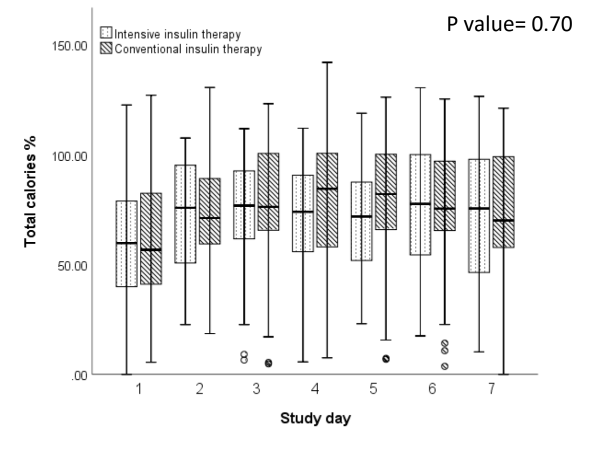	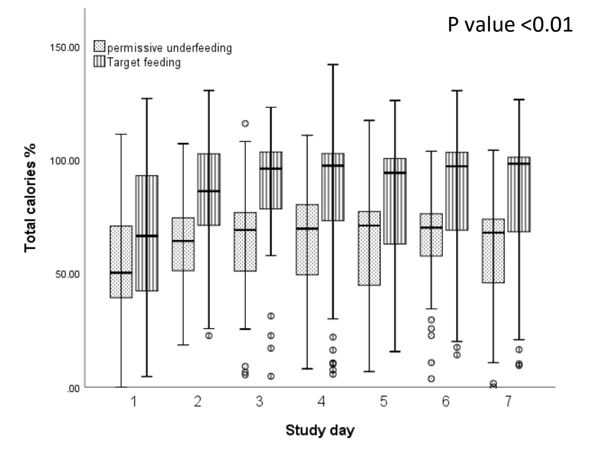
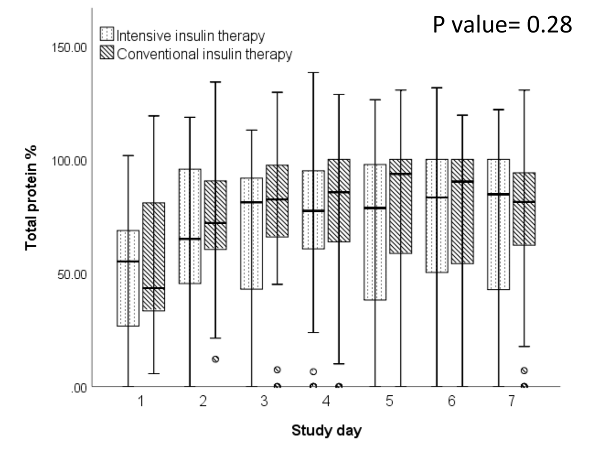	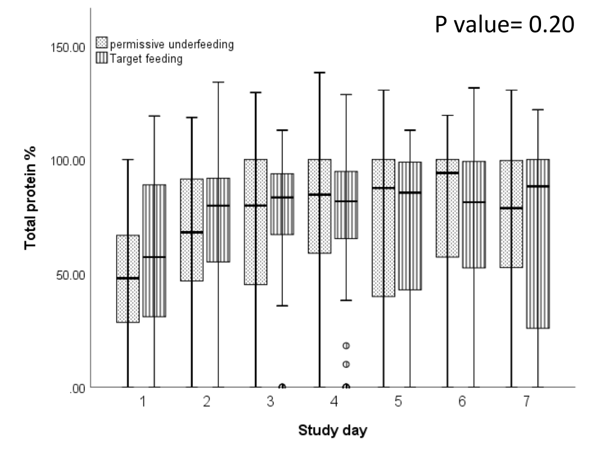

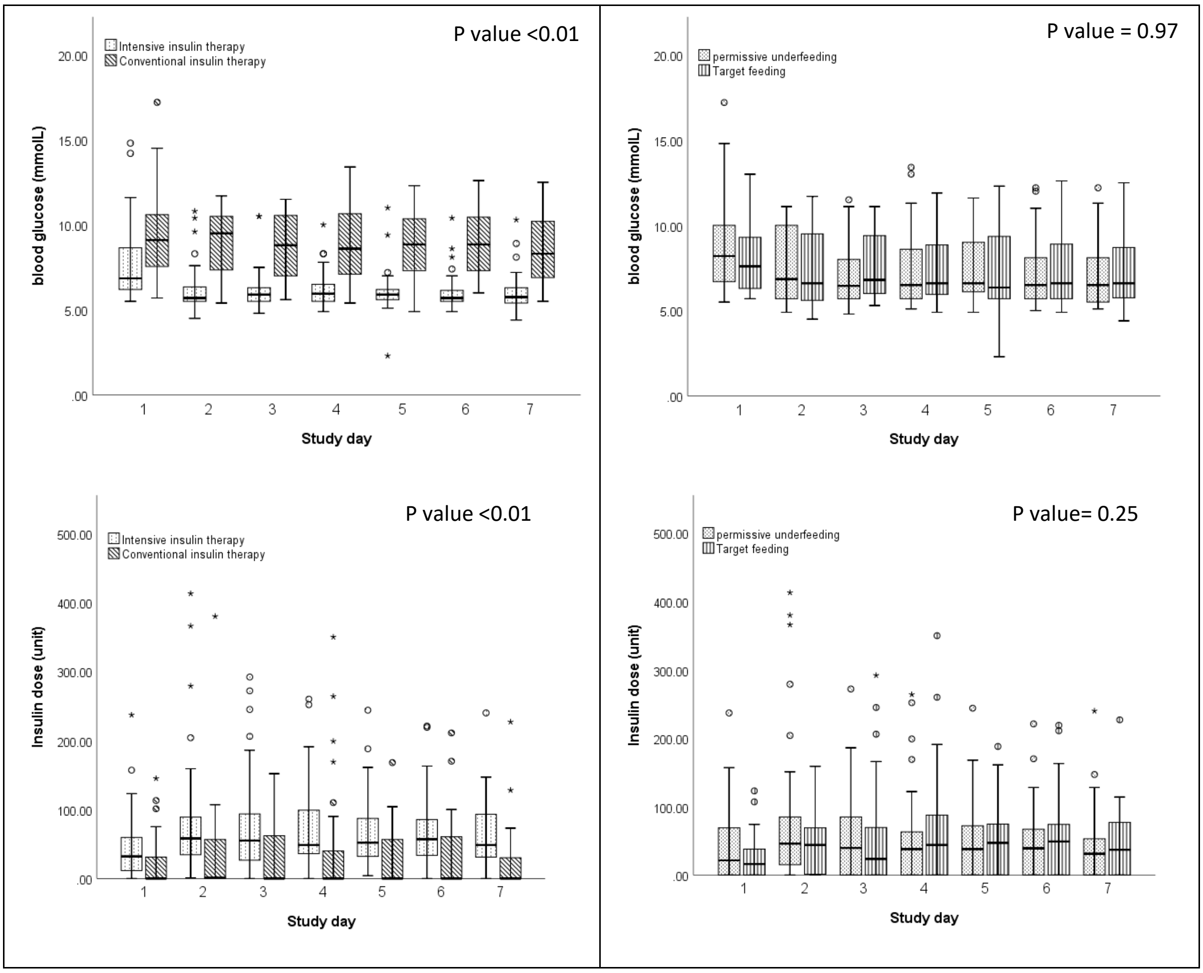
P value <0.01
Intensive insulin therapy
Conventional insulin therapy
blood glucose (mmolL)
Study day
P value = 0.97
permissive underfeeding
Target feeding
blood glucose (mmolL)
Study day
P value <0.01
Intensive insulin therapy
Conventional insulin therapy
Insulin dose (unit)
Study day
P value= 0.25
permissive underfeeding
Target feeding
Insulin dose (unit)
Study day

Figure S2: Plasma MCP-1 (pg/mL), sICAM-1 (ng/mL) and TF (pg/ml) by randomization group at each point of time. Results are presented as Log.

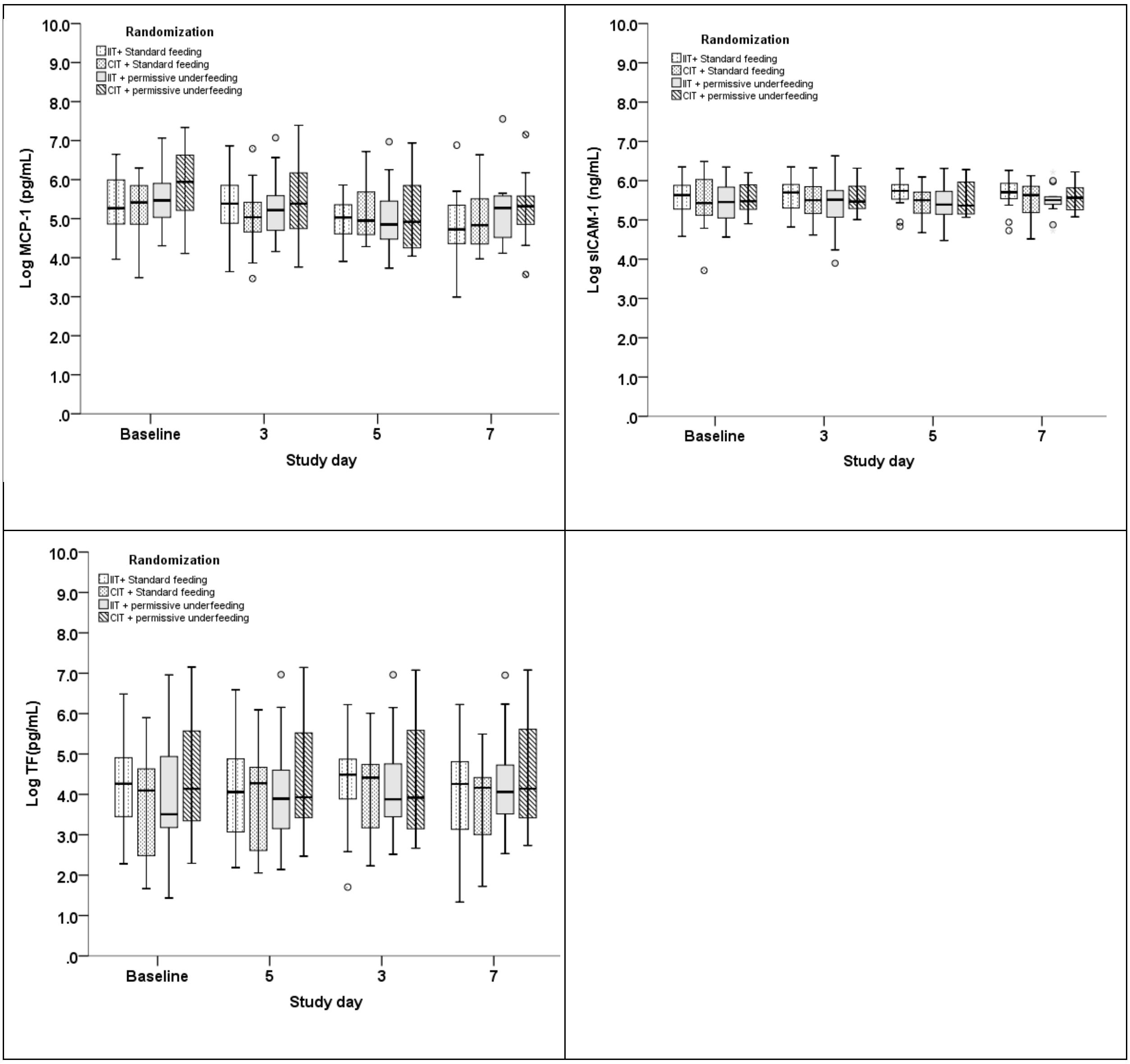

Supplement: Supplementary file 1 [file nutrients-11-00987-s001.pdf]
